# 3D Bioprinting of an Oral Colon Delivery System for Precision Bacteriotherapy

**DOI:** 10.3390/pharmaceutics18060735

**Published:** 2026-06-13

**Authors:** Alessandra Buscarini, Saliha Moutaharrik, Gabriele Meroni, Matteo Cerea, Martina Edith Coldani, Anastasia Foppoli, Luca Palugan, Andrea Gazzaniga, Piera Anna Martino, Alessandra Maroni

**Affiliations:** 1 Sezione di Tecnologia e Legislazione Farmaceutiche “Maria Edvige Sangalli”, Dipartimento di Scienze Farmaceutiche (DISFARM), Università degli Studi di Milano, 20133 Milan, Italy; alessandra.buscarini@unimi.it (A.B.); matteo.cerea@unimi.it (M.C.); martina.coldani@unimi.it (M.E.C.); anastasia.foppoli@unimi.it (A.F.); luca.palugan@unimi.it (L.P.); andrea.gazzaniga@unimi.it (A.G.); alessandra.maroni@unimi.it (A.M.); 2 One Health Unit, Dipartimento di Scienze Biomediche, Chirurgiche e Odontoiatriche, Università degli Studi di Milano, Via Pascal 36, 20133 Milan, Italy; gabriele.meroni@unimi.it

**Keywords:** 3D bioprinting, bacteriotherapy, bacterial viability, oral delivery, colon delivery

## Abstract

**Objectives:** A customizable 3D-bioprinted core-in-shell platform was developed for time-dependent oral colon delivery of live microorganisms. The system conveyed *Lacticaseibacillus paracasei* as a model bacterial species within a monolithic core, which was surrounded by a swellable hydroxypropyl cellulose barrier, imparting a lag phase of programmable duration, and by an enteric outer layer, protecting the dosage form during unpredictable gastric residence. **Methods:** Pastes of different compositions were investigated to shape the core. Core and core-in-shell units were fabricated from digital models using a bioprinter equipped with a high-precision plunger dispenser and pressure-based thermoplastic printhead. The printed units were characterized in terms of mass, dimensions, mechanical properties and release performance using paracetamol as a reference tracer. Bacterial viability was evaluated during screening of the formulation components and after each processing step by manual counting of colony-forming units. **Results:** A mannitol-based formulation was selected for fabrication of the core, offering a favorable balance of printability, physico-technological properties, release behavior and ability to preserve bacterial viability. Two-layer core-in-shell systems were manufactured via a dual-printing operating mode. The desired *in vitro* performance was attained, with no release under acidic conditions, a lag phase in pH 6.8 fluid and a subsequent release profile comparable with that generated by the core as such. Viability studies demonstrated that compounding, core printing, shell deposition and drying did not adversely affect *L. paracasei* survival. **Conclusions:** 3D bioprinting was proved to be a versatile technique for the manufacturing of oral colon delivery systems containing probiotics or live biotherapeutics.

## 1. Introduction

The gut microbiota constitutes a complex, dynamic ecosystem of bacteria, fungi, viruses, and protozoa that interacts mutually with the host [1]. The relevant composition varies considerably along the gastrointestinal tract, with the colon representing the most densely populated and metabolically active region. Imbalance in this community, commonly termed dysbiosis, has been associated with the pathogenesis of several chronic conditions, not limited to the gastrointestinal tract [2,3]. Particularly, colonic pathologies affected by dysbiosis encompass inflammatory bowel disease (IBD) such as Crohn’s disease and ulcerative colitis [4]. The global incidence of IBD and other intestinal disorders, including colorectal cancer (CRC), irritable bowel syndrome (IBS) and diverticular disease, continues to rise, fueled by Westernized diets, aging populations and sedentary lifestyles [5,6,7]. It has also been reported that chronic inflammation markedly elevates CRC risk [8]. Hence, there is growing interest in therapeutic strategies that may restore microbial balance. Probiotics, defined by the FAO/WHO (2001) as “live microorganisms that, when administered in adequate amounts, confer a health benefit on the host”, are a cornerstone of such strategies [9]. *Lactobacillus*, *Bifidobacterium*, and *Streptococcus* are frequently used *genera* alongside yeasts such as *Saccharomyces boulardii*. These microorganisms exert several beneficial effects, mainly consisting of immune modulation, enhancement of epithelial barrier integrity, production of antimicrobial compounds and competitive exclusion of pathogens [10]. However, orally administered bacteria are required to reach the large bowel in a viable form. A first, major challenge lies in preserving viability throughout production and storage. Indeed, this may be impaired in solid dosage forms such as tablets, where mechanical compaction forces and locally elevated temperatures, beyond oxygen and moisture, can reduce survival rates [11,12]. Certain binders, lubricants and disintegrants may interact adversely with probiotic cells [13]. Coating processes aimed at modified release could also involve threatening conditions. Large-scale production would bring about further stability issues related to supply chain and long-term storage [14]. Substantial declines in viable counts are often observed even during refrigerated storage, with numbers dropping below the therapeutic threshold prior to expiry [15,16,17]. Following intake, bacteria are exposed to the acidic gastric fluid, bile salts and digestive enzymes in the upper gastrointestinal tract, which may seriously harm their viability and colonization potential [18]. Moreover, conventional “one-size-fits-all” products would overlook intra- and inter-subject differences in gut microbiota composition, limiting implementation of precision bacteriotherapy or personalized supplementation treatments that meet the highly diverse needs of each individual patient or consumer [19]. Therefore, for tailored and effective probiotic administration, the carrier dosage form should not only maintain cell viability throughout all manufacturing, storage and usage phases, but also release its bioactive payload selectively into the colon and be amenable to customization in terms of type and dose of bacteria delivered.

Three-dimensional (3D) printing, encompassing a range of additive manufacturing techniques, has recently been leveraged for the fabrication of advanced oral colon delivery systems, mainly based on pH-dependent targeting, containing mesalazine, budesonide, camptothecin, oxalilplatin or different model drugs [20,21,22,23,24,25,26]. To this end, fused deposition modeling (FDM) has primarily been exploited.

Among 3D printing techniques, bioprinting employs bio-inks that are deposited layer by layer to achieve functional biocompatible structures [27,28,29]. Besides its broad appeal for biomedical uses, it holds potential for novel drug delivery applications. Interestingly, bioprinted probiotic formulations for colonic release have already been described [30,31]. Indeed, such a technique could fulfill the previously discussed requirements, allowing for extemporaneous fabrication of custom-designed formulations, avoiding the high compaction pressures and elevated temperatures of conventional processes, namely tableting and coating, facilitating the use of bacteria-friendly formulations and providing geometries that can help protect bacteria from the environment [32]. Like other 3D printing modes, bioprinting would also enable prototyping for relatively larger-scale manufacturing [33,34]. Reported cases of real-world deployment of 3D printers, also in GMP-compliant configurations, would support the possibility of point-of-care, on-demand manufacturing of personalized dosage forms [35].

Based on these premises, the present study was aimed at developing a 3D-bioprinted oral colon delivery system housing, shielding and conveying live microorganisms for bespoke bacteriotherapy applications. A single-parameter formulation strategy for colonic release was preferred over hybrid ones in light of the simpler composition and design to implement in this study. Particularly, a time-dependent approach was followed, relying on the relatively consistent small intestinal transit time of dosage forms, as supported by broad and diverse datasets [36,37,38]. Accordingly, the delivery system was conceived as a reservoir form, embedding the bacteria within a monolithic solid core. The core was surrounded by a swellable hydrophilic polymer barrier, typically based on cellulose derivatives subject to hydration, erosion and dissolution processes upon contact with intestinal fluids over a programmable timespan. Externally, an enteric layer was needed to preserve the inner compartments during unpredictable gastric residence, thus allowing for site-targeted release. Notably, the two-layer core-in-shell configuration pursued represented a challenging goal to achieve via 3D bioprinting.

*Lacticaseibacillus paracasei*, formerly *Lactobacillus paracasei*, was employed to address the formulation and fabrication of the dosage form and assess the relevant impact on cell viability. *L. paracasei* is a Gram-positive member of the lactic acid bacteria group, isolated from the gastrointestinal tract of humans and animals, fermented foods and plant-based substrates [39]. The optimal growth temperature is in the 10–37 °C range, with viability declining sharply beyond it and ceasing above 40 °C. Storage at a refrigerated temperature (4 °C) enhances survival rates [40]. While such overall features posed processing and stability challenges, formulation development could still have been undertaken in the experimental setting where the study was conducted, making the bacterium an appropriate model for this preliminary investigation. Over the past decade, *L. paracasei* has garnered attention due to a broad spectrum of health-promoting activities [41,42]. Its use has also been explored in the management of gastrointestinal disorders including diarrhea [43], IBS [44] and IBD [45]. A long-standing safety profile and role in maintaining mucosal and microbial homeostasis within the host microbiota have been documented [46].

## 2. Materials and Methods

### 2.1. Materials

Crospovidone (Kollidon^®^ CL, BASF Italia S.p.A., Cesano Maderno, Italy); hydroxypropyl cellulose (HPC, Klucel™ LF, Ashland, Milan, Italy); hydroxypropyl methylcellulose (HPMC) grades Methocel^®^ E5 Premium LV and Methocel^®^ E50 Premium LV (Colorcon, Dartford, UK); hydroxypropyl methylcellulose acetate succinate (HPMCAS, AQOAT^®^, Shin-Etsu Chemical Co., Tokyo, Japan); lactose (Lactochem^®^ Crystals, DFE Pharma, Goch, Germany); mannitol grades Mannogem^®^ Powder and Mannogem^®^ XL (SPI Pharma, Septèmes-les-Vallons, France); colloidal silicon dioxide (Aerosil^®^ 200, Evonik Italia S.p.A., Pandino, Italy); paracetamol fine powder (Rhodia, Bollate, Italy); poly(vinyl acetate) and povidone blend (Kollidon^®^ SR, BASF Italia S.p.A.); polyethylene glycol 400 and 1500 (PEG 400 and PEG 1500, Clariant Masterbatches, Milan, Italy); sodium lauryl sulfate (Evonik Italia S.p.A.); sodium starch glycolate (Explotab^®^ CLV, JRS Rettenmaier Italia, Castenedolo, Italy); triethyl citrate (TEC, Sigma-Aldrich, Milan, Italy).

### 2.2. Methods

#### 2.2.1. Fabrication of the Delivery System

*Lacticaseibacillus paracasei* CNCM I-1572 was isolated from a commercially available probiotic product and cultivated in de Man–Rogosa–Sharpe medium (MRS, Thermo Scientific™, Basingstoke, UK). A total of 50 µL of a pure frozen culture of *L. paracasei* CNCM I-1572, preserved in 25% *v*/*v* glycerol, was thawed, plated on MRS agar and incubated anaerobically (Anaerogen, Thermo Scientific™) at 37 °C for 72 h. A well-isolated colony was then transferred to 25 mL of MRS broth and incubated under the same conditions. Following centrifugation at 4000× *g* for 30 min at room temperature, the supernatant was discarded, and the pellet was resuspended in fresh phosphate-buffered saline. After a second centrifugation step under identical conditions, the pellet was collected and used for incorporation into the formulation. For bacterial enumeration, 1 mL of the original culture was serially diluted 10-fold up to 10^−9^, and 50 µL of each dilution was plated in triplicate on MRS agar. Plates were incubated anaerobically at 37 °C for 72 h, and colony-forming units (CFUs) were manually counted to determine the pellet bacterial concentration.

Pastes to be extruded for core fabrication were prepared using a mechanical stirrer equipped with blades (RW 18, Janke&Kunkel GmbH & Co. KG, Staufen im Breisgau, Germany) operated at 150–200 rpm. Paracetamol, employed as an analytical tracer, was dissolved in distilled water, and the selected excipients were sequentially added. When a homogeneous suspension or a clear solution was obtained, colloidal silica was gradually incorporated, followed by HPMC. Where applicable, a superdisintegrant was incorporated in successive aliquots. The paste developed for probiotic incorporation was refrigerated at 4 °C for 16–24 h, and bacterial pellet was finally added at a concentration of 1% of the total mass. For the fabrication of shells, all materials except for plasticizers were kept in an oven at 40 °C for 24 h prior to use. The polymeric formulations were prepared in a mortar by adding the plasticizer to the dry polymer under continuous manual mixing.

The bioprinting processes were performed using a 3D Discovery™ Gen. 5 bioprinter (RegenHU, Villaz-Saint-Pierre, Switzerland) equipped with a High-Precision Plunger Dispenser (HPD-100) and a pressure-based thermoplastic printhead (DD-135H). Designs were created using AutoCAD Version R24.1 (Autodesk, San Francisco, CA, USA) and model-refinement software (BioCAM Version 1.1.0.6., RegenHU). All printed specimens were kept in a desiccator for 24 h prior to further testing.

When bacteria were handled, compounding operations were performed under a fume hood, and the employed equipment as well as glassware were previously treated with 75% *v*/*v* ethanol.

#### 2.2.2. Characterization of the Printed Units

Printed samples were characterized in terms of mass, dimensions, mechanical characteristics, release behavior and bacterial viability, as allowed by the limited batch sizes. Mass and dimensions were expressed as mean values ± standard deviation.

Particularly, cores were characterized for mass (analytical balance BP211D, Sartorius Italy S.r.l., Varedo, Italy; *n* = 10), height and diameter (digital micrometer, Mitutoyo Italiana S.r.l., Lainate, Italy; *n* = 10), crushing strength (tablet hardness tester TBH30, Erweka GmbH, Langen, Germany; *n* = 3) and friability (friabilometer TA3R, Erweka GmbH; *n* = 6). Core-in-shell specimens, one- or two-layer, were characterized for mass *(n* = 10), height and diameter *(n* = 10). Release tests (*n* = 3) were carried out by an adapted disintegration apparatus (DT3, Sotax S.r.l., Milan, Italy). This testing mode was preferred over conventional dissolution in that the typical hydrodynamics was proved effective in preventing adhesion of the swollen hydrophilic polymer of the inner layer to the internal container wall, thus overcoming the data reliability issues observed with conventional dissolution equipment [47,48]. For the test, a single unit was inserted into each basket-rack assembly of the apparatus, and fluid samples were automatically withdrawn for analysis at successive time points. The tracer released was assayed by spectrophotometer (Lambda 35, PerkinElmer^®^ Italia, Milan, Italy) at 248 nm. Next, 800 mL of phosphate buffer (PB) pH 6.8 at 37.0 ± 1.0 °C was used as the release medium for cores and one-layer core-in-shell systems, while 0.1 N hydrochloric acid (HCl) solution for 2 h and afterward PB pH 6.8 were used with two-layer core-in-shell systems. Lag time was calculated as the time to 10% release in PB pH 6.8 (t10%) by linear interpolation of the data immediately preceding and following this release percentage [49,50].

#### 2.2.3. Viability Studies

To assess the viability of *L. paracasei* after compounding, processing and short-term storage, samples (*n* = 3) were collected at multiple time points. All were weighed, and mass values were recorded to enable an initial 1:10 dilution in sterile 0.85% sodium chloride (NaCl) solution. Specifically, dilutions were carried out in a 10-fold series starting from each sample homogenized in 9 volumes of sterile 0.85% NaCl solution (10^−1^) up to 10^−9^. Subsequently, 50 μL aliquots from each dilution were plated in triplicate on MRS agar and incubated at 37 °C for 72 h, after which CFUs were enumerated by manual counting. Data were processed by BioRender (www.biorender.com; accessed 20 April 2026), and a Kruskal–Wallis nonparametric test was run to assess statistically significant differences (*p* < 0.05).

## 3. Results and Discussion

### 3.1. Development of the Core 

The formulation of the core was developed in the form of a paste, i.e., a semisolid substrate with a relatively high solid content, suitable for printing. This would involve acceptable inherent extrudability on the one hand and, on the other hand, ability to yield mechanically stable constructs having satisfactory shape fidelity and physico-technological characteristics, consistent with compressed tablets. A previous formulation, reported to provide a reasonable balance of such different aspects, served as a basis for setting up the composition of the core through a trial-and-error approach [51]. Prior to addition of the cultured bacteria, multiple variants were explored to progressively adapt the paste to the specific requirements of the delivery system. Particularly, the printed core was expected to undergo sufficiently prompt release in contact with aqueous fluids and, therefore, not to have a major impact on the overall *in vitro* performance, which was intended to be governed merely by the functional layers. To monitor the behavior of cores, a tracer small molecule was added as a reference, and a target of 70% release within 30 min was set. In line with previous analogous delivery systems, paracetamol was used as the analytical tracer given its well-known solubility, solid-state properties, ease of UV detection and lack of major reported detrimental effects on bacteria [52,53]. An insoluble component (Kollidon^®^ SR) was first employed as a filler for structuring purposes, while fumed silica and HPMC were incorporated as viscosity-enhancing agents (Table 1). Through preliminary printing tests, the semi-solid paste was evaluated on extrusion, assigning defined values to each process parameter (Table 2). In more detail, infill density was initially kept relatively low to aid post-processing drying, and a 45° orientation pattern relative to the long axis was imparted to counteract shrinkage of the printed dosage forms. Aiming to achieve a sufficiently cohesive and mechanically resistant structure, a 5% overlap was defined between the internal mesh and the surrounding perimeter. Furthermore, fixed dimensions were established for the oval-shaped core: 7.8 mm, 14.0 mm and 3.9 mm, along the *x*-, *y*- and *z*-axes, respectively.

**Figure 1:**
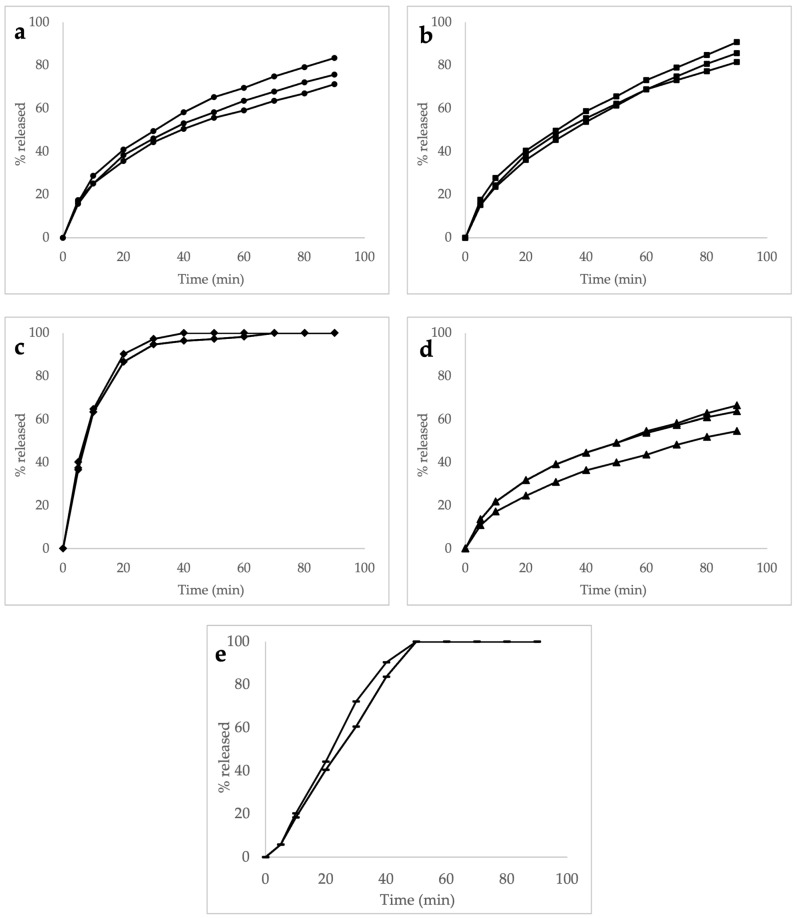
Individual release profiles of (**a**) F1, (**b**) F3, (**c**) F7, (**d**) F8 and (**e**) F9 prototype cores.

With the starting formulation (F1), printing proceeded without disruption: 10 units were fabricated sequentially, and each of them was within ±10% of the average mass meeting the pharmacopeial uniformity limits for galenical preparations. After drying, the units retained a consistent shape with an average dry mass of 169 ± 6 mg. Dimensional deviations from the CAD model were anisotropic. Expansion along the short diameter, coupled with concomitant reduction in the long diameter and height, would suggest gravitational spreading during the solvent evaporation phase due to partial failure of vertical support. Nonetheless, the units were sufficiently hard to handle. Friability testing showed only 0.1% weight loss, indicating minimal dusting. Subjected to the crushing test, the printed cores were elastically deformed before breaking. Crushing strength, while being variable, averaged 337 N. Slow release profiles were obtained, with the rate decreasing over time. Approximately 70% of the drug was released within 75 min, and solid portions detached from the printed units were still present in the medium by the end of the test (Figure 1a).

To improve the performance of the printed cores, two different strategies were implemented: the insoluble filler Kollidon^®^ SR was either replaced with a soluble one, or combined with a superdisintegrant.

In the former instance, it was initially replaced with 50% mannitol (Mannogem^®^ Powder, F2). Such a compound was expected to undergo partial dissolution upon contact with biological fluids, weakening the overall structure and creating interconnected pores within the dosage form. Following this change in composition, however, the printing process failed to yield prototypes of the desired mechanical quality.

The use of Mannogem^®^ XL was attempted in place of Mannogem^®^ Powder (F3). Mannogem^®^ XL is a different commercially available mannitol product showing higher particle size, lower apparent density and larger specific surface area compared with the standard grade previously employed, consistent with the inherent porous structure generated through spray-drying [54,55]. Indeed, its claimed applications include orally disintegrating and effervescent tablets. Hence, Mannogem^®^ XL was assumed to possibly aid in the formation of a mechanically stable structure on the one hand, and promote its interaction with aqueous media on the other hand. The resulting paste enabled smooth flow on extrusion, increased adhesion between layers and achievement of complete printed units. After drying, the mean weight was 250 ± 35 mg, and 2 units fell outside the ±10% mass variability acceptance limit. Both diameters exceeded the CAD targets and showed greater spread along the *x*-axis. Moreover, *in vitro* release was still excessively slow, consistent with a disintegration time >2 h (Figure 1b).

Therefore, Kollidon^®^ SR was fully replaced with a soluble filler, such as lactose or mannitol. Pastes based on lactose (Lactochem^®^ Crystals) (F4) or Mannogem^®^ Powder (F5) exhibited poor flow properties and unexpected solid/liquid phase separation when subjected to extrusion pressure, with consequent cartridge clogging caused by dense packing of the solid fraction. This was attributed to a lower ability of the dissolved fillers to maintain a homogeneous paste composition under shear stress.

In the Mannogem^®^ Powder formulation (F5), the percentage amount of colloidal silica was increased while slightly decreasing the water content to enhance the overall viscosity of the extrudable paste and possibly reduce liquid squeeze-out (F6). Although such goals were met, the process remained challenging, allowing for only few incomplete prototypes to be printed.

**Figure 2:**
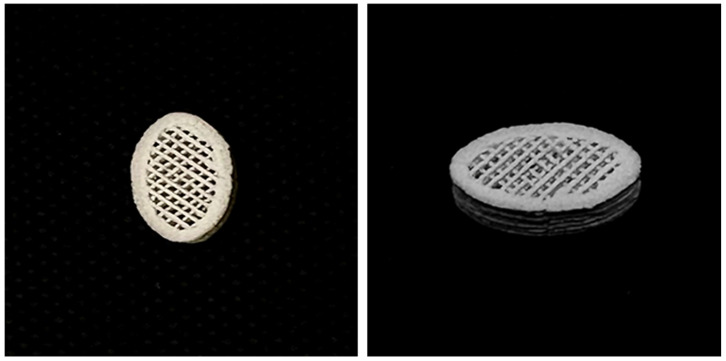
Side (**right**) and top-down (**left**) photographic views of F7 prototype cores.

On the other hand, the use of Mannogem^®^ XL in place of Mannogem^®^ Powder led to a more smoothly processable paste (F7): 10 units were printed in sequence with only brief pauses, exhibiting a reproducible dry mass of 170 ± 5 mg (Figure 2). Compared with F1, these spread slightly in the *x*–*y* plane while shrinking along the *z*-axis. Crushing tests revealed a rigid, brittle nature. Indeed, the printed cores fractured into small fragments rather than deforming elastically, yet friability remained acceptable at 0.4%. Release profiles of F7 are shown in Figure 1c.

Over 90% of the tracer was released in 25 min, approximately 6-fold faster than previously observed, meeting the goals set in this respect. Moreover, the individual profiles were nearly superimposable. During the *in vitro* test, the dosage forms began to disintegrate in early stages of exposure to the medium. The internal mesh disintegrated first, whereas more compact outer perimeters took longer. The time required for complete disintegration of the units ranged from 15 to 25 min.

To promote interaction with aqueous fluids, insoluble Kollidon^®^ SR was combined with a superdisintegrant instead of being replaced with a soluble filler. Two candidates were screened, namely sodium starch glycolate (Explotab^®^ CLV) and crospovidone (Kollidon^®^ CL). Both were incorporated last to limit early swelling. In the Kollidon^®^ CL-containing formulation (F8), the polymer swelled immediately upon contact with residual water, increasing the volume of the paste, coarsening its texture and reducing surface gloss. Water uptake by the superdisintegrant made the paste more viscous, yet flow through the nozzle remained continuous. Although inter-layer adhesion appeared weaker, this issue was partially offset by the expansion of freshly extruded filaments that helped maintain structural consistency. After drying, the tablets showed a mean mass of 210 ± 13 mg, pointing out good reproducibility. Dimensional data followed the trend observed in the previous formulations: the short diameter slightly increased, and the long diameter decreased with respect to the CAD target. Despite the superdisintegrant added, *in vitro* testing revealed no acceleration in drug release compared with F1 (Figure 1d).

With Explotab^®^ CLV, printing turned out to be feasible, and the dried units had a mean mass of 156 ± 19 mg (F9). In contrast to earlier formulations, all three dimensions were below the CAD specifications, indicating appreciable shrinking upon water removal. A clear improvement was observed in the performance of F9 (Figure 1e). Indeed, the time to complete disintegration ranged from 25 to 40 min, and the release rate was higher than with F1 or F8, reaching approximately 70% in 30 min.

Based on the overall results, either replacement of Kollidon^®^ SR with soluble filler Mannogem^®^ XL or incorporation of superdisintegrant Explotab^®^ CLV turned out to provide feasible printing and printed cores having acceptable physico-technological as well as release characteristics. The formulations showing a favorable balance of printability, on the one hand, and quality as well as *in vitro* performance on the other hand, namely F7 and F9, were selected as core candidates for *L. paracasei* CNCM I-1572 delivery. A staged study was thus conducted to rule out any negative impact of the relevant components on bacterial viability. Starting from a plain probiotic suspension, each excipient was added sequentially, and the number of CFUs was measured after addition and incubation, thus allowing any individual and cumulative effects to be recorded. The starting suspension had a turbidity value of McF = 13 (McFarland Standard), corresponding to a concentration of 1.2 × 10^10^ CFU/mL. With regard to the Mannogem^®^ XL-based formulation, incorporation of the polyol increased the viable count to above 10^12^ CFU/mL, and the subsequent incorporation of Methocel™ E5 also raised it slightly to 6.3 × 10^12^ CFU/mL, pointing to a possible protective effect of such substances. Conversely, the addition of fumed silica resulted in a 2-log reduction, lowering viability to about 6.0 × 10^8^ CFU/mL. Although unexpected, this loss could be attributed to the known desiccating action of fumed silica. Partial removal of free water may have weakened the bacteria and made them more susceptible to heat damage [56]. With the formulation based on Explotab^®^ CLV, Kollidon^®^ SR was first added to the bacteria, which eradicated detectable CFUs entirely. Explotab^®^ CLV itself, when evaluated in the absence of Kollidon^®^ SR, did not appreciably diminish bacterial counts. Given the detrimental antibacterial effect of Kollidon^®^ SR, F9 was discarded, and only F7 was advanced to the subsequent steps of the study.

### 3.2. Development of Core-in-Shell Systems

**Figure 3:**
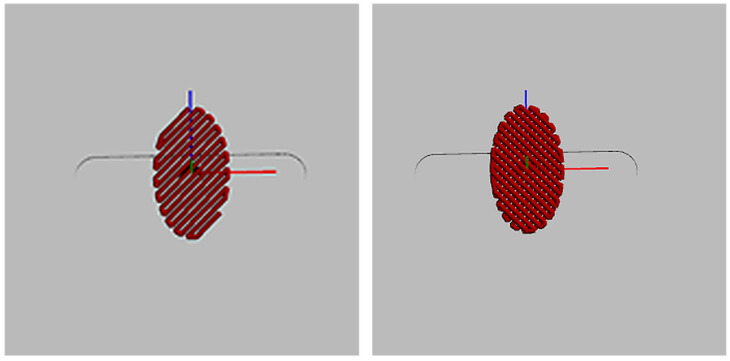
Top-down BioCAM views of the layered structure printed during fabrication of the core.

To enable time-dependent colon delivery, the polymer shells based on HPC and, respectively, HPMCAS needed to be incorporated into the dosage form. F7, which emerged as the formulation candidate best balancing processability, quality, performance and preservation of bacterial viability, was used as the core for this purpose. A dual-printing system was employed: the inner core was fabricated making use of the HPD-100 printhead, and the outer shell was deposited by means of the DD-135H one. An updated design was required for the multilayered delivery platform. For precise matching with the outer layers, the core was slightly downsized and, given the structural support offered by the polymer shell, its outer perimeter was omitted. Moreover, the infill density was raised to increase the total load (Figure 3). Dimensions along the *x*-, *y*- and *z*-axes were set to 7.0 mm, 12.6 mm and 3.5 mm for core, 9.4 mm, 15.1 mm and 5.8 mm for one-layer core-in-shell and 11.6 mm, 18.6 mm and 8.5 mm for two-layer core-in-shell units, respectively. The printing parameters were tuned to ensure full compatibility of the core with the shells (Table 3).

**Figure 4:**
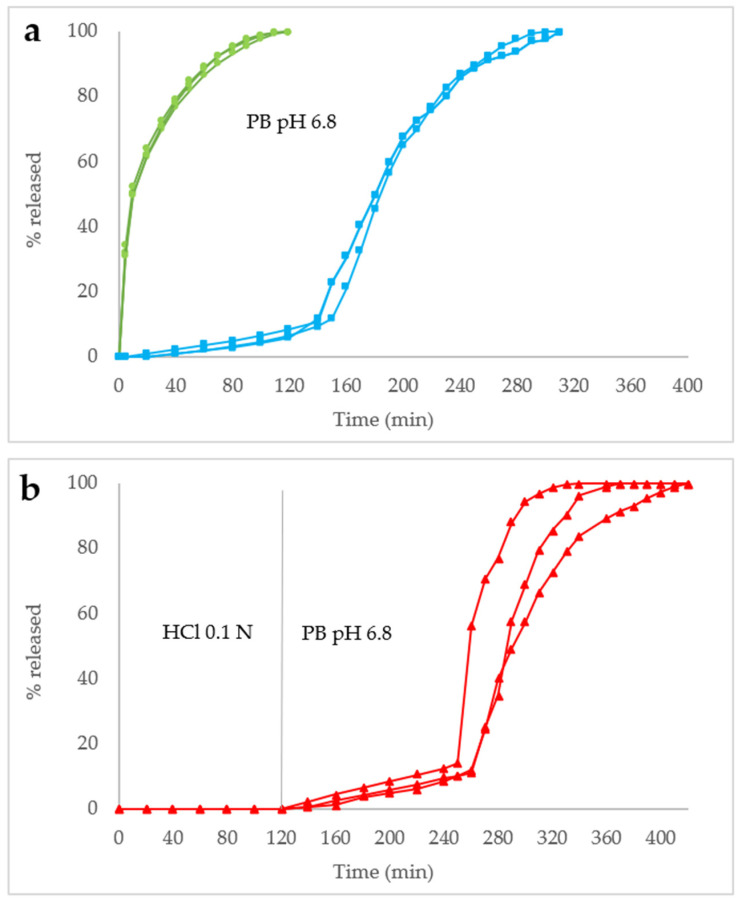
Individual release profiles of (**a**) cores (circles) and one-layer core-in-shell systems (squares), and (**b**) two-layer core-in-shell systems (triangles).

Ten prototypes were printed and evaluated. Because of the modified settings, the cores exhibited a lower mass than the earlier ones (119 ± 7 mg). Dimensional analyses also showed a modest reduction in height and long diameter. Removing the perimeter walls weakened the units, making them more prone to print defects and shrinkage-driven collapse during drying. Friability testing confirmed the observed fragility: mass loss reached 2.8%, substantially higher than in previous batches. Crushing strength averaged 46 N, indicating the rigid, brittle characteristics of F7. The new cores exhibited a slower release than previously observed (Figure 4a). This was attributed to the altered print architecture: raising the infill, water penetration was restricted by the denser structure obtained. All curves closely overlapped, highlighting good run-to-run reproducibility.

**Figure 5:**
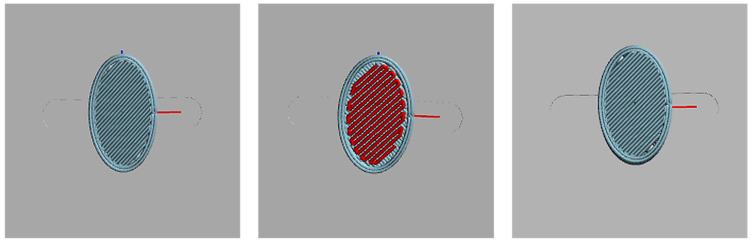
Top-down BioCAM views of the core-in-shell structure printed in the dual-printhead mode: firstly, deposited base (**left**), subsequently, co-printed core and inner shell (**center**) and, finally, applied top layer (**right**).

To introduce a programmable lag phase before release, HPC, a soluble cellulose ether, was employed for fabrication of the inner shell. Particularly, a low-viscosity grade, Klucel™ LF, was selected due to its favorable rheological and thermoplastic properties, already known to support extrusion-based 3D printing [57,58,59]. The polymer was plasticized with 15% PEG 1500, and the mixture was extruded at 165 °C. Viscosity was manageable, enabling precise layer deposition and shape fidelity. Using the dual-printhead operating mode, the base of the HPC shell was deposited first, after which the inner core and the lateral shell wall were simultaneously printed by means of the HPD-100 and DD-135H printheads, respectively. The construct was completed by deposition of a capping outer layer (Figure 5).

**Figure 6:**
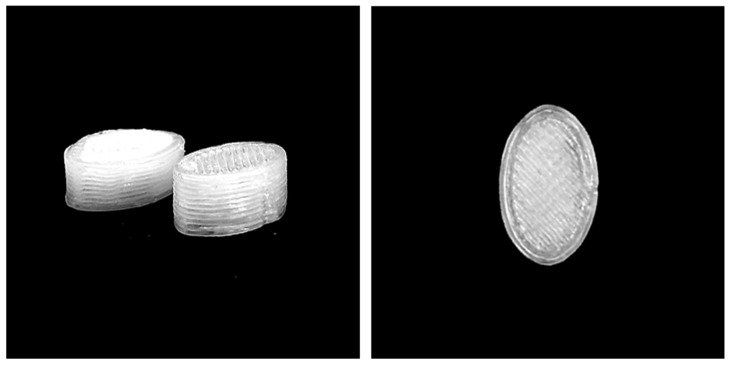
Top-down (**right**) and side (**left**) photographic views of two-layer core-in-shell systems with (right-hand and central units) and without (left-hand unit) final capping layer.

Once extruded, the formulations cooled to room temperature within seconds and solidified, retaining the shape and size imparted. Dimensions were closer to the CAD model compared to the uncoated cores, likely due to the rapid solidification of the shell material that reduced post-printing shrinkage. The print run proceeded without interruptions, yielding 10 units in less than 20 min. The mean mass was 643 ± 90 mg. While mass variability exceeded the compendial acceptance limits, this was not observed with the core as such, suggesting a lower impact on dosing accuracy. Representative images of printed one-layer core-in-shell prototypes are shown in Figure 6.

**Figure 7:**
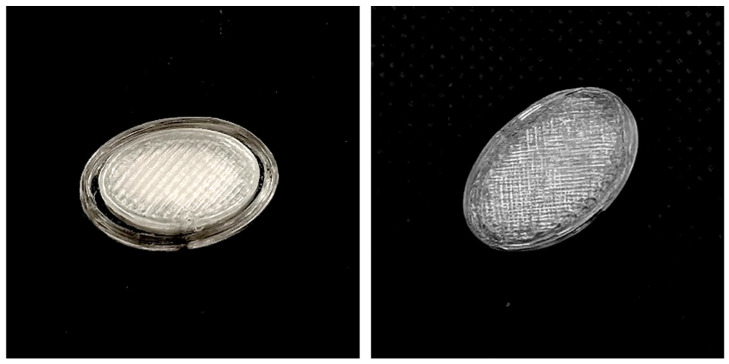
Top-down photographic views of two-layer core-in-shell systems with (**right**) and without (**left**) final capping layer.

Three systems were evaluated in terms of *in vitro* behavior (Figure 4a). After a lag time (t10% = 150 min), each unit entered a release phase, which was superimposable to that generated from the cores as such. Besides the applied HPC barrier, intended to delay the onset of release throughout small intestinal transit of the dosage form, an enteric layer was required to achieve time-controlled site-selective release into the colon. HPMCAS (AQOAT^®^), a pH-responsive cellulose derivative widely used in oral dosage forms for gastroresistance purposes, was selected to this end because of its proven suitability for extrusion-based 3D printing [60,61]. Given the dual-printhead set available, fabrication of the core and of the overlapping HPC and HPMCAS layers required a double processing run. The enteric outer barrier was thus printed in two stages: approximately half of the shell structure was first deposited by the DD-135H printhead, creating a self-supporting cavity. Printing was then paused to encase a pre-formed one-layer core-in-shell unit, and finally resumed to complete and seal the outermost enteric layer. HPMCAS was blended with 20% TEC, used as a plasticizer. The mixture was extruded at 155 °C, which enabled consistent flow of the softened material, adequate shape retention and reproducible formation of printed units. Despite this staggered procedure, the process was carried out smoothly and yielded, within 10 min, 10 intact prototypes with a mean mass of 750 ± 90 mg, suitable for characterization. Mass variability was still outside the compendial acceptance limits. However, dimensions were quite close to the CAD file, indicating that shape was retained over time, and major post-printing shrinkage phenomena were avoided. An example is displayed in Figure 7, whereas the release profiles from the two-layer core-in-shell systems obtained are shown in Figure 4b. The units remained intact after 2 h in the acidic medium with no detectable release. Upon transfer to phosphate buffer pH 6.8, a lag time of around 130 min was observed, consistent with that of one-layer core-in-shell systems tested in the same fluid. Interestingly, data previously collected from analogous reservoir systems, provided with a low-viscosity hydrophilic cellulose derivative coating, indicated generally more extended delays *in vivo* than *in vitro* [38,62]. The lag phase was followed by a rapid release that closely matched the release profile of the core as such.

**Figure 8:**
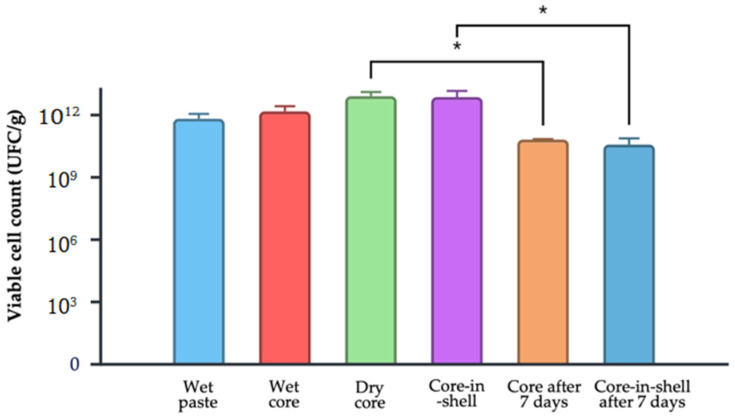
Viability of *L. paracasei* in different samples (vertical bars indicate standard deviation; * indicates statistically significant differences).

The printed delivery platform under development was loaded with *L. paracasei*, conveyed within the core of the dosage form. To confirm applicability of such a technology to the model probiotic, a viability assessment was performed after different processing steps. In more detail, the samples tested encompassed: (*i*) probiotic-containing paste intended for core printing, (*ii*) core immediately after printing, (*iii*) core after overnight drying, (*iv*) one-layer core-in-shell system immediately after extrusion and, finally, (*v*) core and one-layer core-in-shell system stored for 7 days at 4 °C to explore short-term viability, consistent with the intended use of an extemporaneous formulation for personalized treatments (Figure 8). The paste after compounding showed a viable count of 6.7 × 10^11^ CFU/g. The extruded core reached approximately 1.5 × 10^12^ CFU/g, indicating that the formulation itself could provide a favorable environment for the cells, and extrusion pressure would not negatively impact on viability. Interestingly, dried cores exhibited a viable count of 8.1 × 10^12^ CFU/g, pointing to preservation of the bacteria during the drying step possibly due to reduced metabolic stress. With the core-in-shell system, viability remained high at 7.4 × 10^12^ CFU/g, confirming that addition of the HPC layer did not hinder bacterial survival immediately after processing. Following short-term storage, however, both the core and core-in-shell samples showed a reduction in viable counts. Indeed, values of 3.8 × 10^10^ CFU/g and 6.6 ×10^10^ CFU/g were found, respectively. Although the data recorded remained largely above the range that is commonly considered adequate for effective probiotic formulations, this result strengthens the need for extending the viability study over a longer time span.

## 4. Conclusions

Feasibility of 3D bioprinting in the manufacturing of core-in-shell oral dosage forms was explored for time-dependent colon delivery of live microorganisms. Different extrudable paste formulations were developed to form the core, balancing printability, physico-technological quality, release kinetics and lack of major harmful effects on *L. paracasei* CNCM I-1572. Particularly, drug release performance meeting the set goals was achieved from F7, based on Mannogem^®^ XL, and F9, containing Kollidon^®^ SR and Explotab^®^ CLV. However, while staged viability testing indicated that Mannogem^®^ XL, HPMC and Explotab^®^ CLV supported or enhanced survival of bacteria, Kollidon^®^ SR reduced viable counts. Starting from F7, a dual-printhead manufacturing approach enabled fabrication of a core-in-shell system with adjustable internal geometry and an HPC-based functional layer. This provided reproducible *in vitro* lag times in the 130–150 min range before a rapid release phase closely matching the behavior of the core devoid of shells. Subsequent application of a pH-responsive HPMCAS enteric layer via a two-step printing process successfully imparted compendial gastroresistance properties, as needed according to the time-dependent colon delivery approach followed. Both core and core-in-shell constructs exhibited high bacterial viability immediately after processing and drying, with counts on the order of 10^10^ CFU/g after 7 days of storage at 4 °C, demonstrating that the printing process itself did not adversely affect the probiotic under the operating conditions in use. The obtained results would support application of 3D bioprinting as a versatile strategy for the manufacturing of oral delivery systems with both modified release and live biotherapeutic capabilities. By combining the advantages of solid oral dosage forms, biological requirements for live bacterial cells and customization demanded by precision therapy, the proposed delivery platform may be leveraged for treatments restoring each individual’s gut microbial balance in intestinal and extra-intestinal disorders. This potential will need to be confirmed through *in vivo* validation along with more solid evidence on stability and translation into practice. While deepening investigation into the effects of common excipients on probiotic viability, future studies will also address feasibility of the technology with different bacterial strains and diverse polymer mixtures aimed at hybrid colon targeting strategies.

## Figures and Tables

**Table 1:** % composition of formulations under investigation.

	F1	F2	F3	F4	F5	F6	F7	F8	F9
Distilled water	54.6	54.6	54.6	54.6	54.6	52.1	54.6	52.1	52.1
Paracetamol	3.0	3.0	3.0	3.0	3.0	3.0	3.0	3.0	3.0
Kollidon^®^ SR	24.2	12.1	8.1	-	-	-	-	23.2	23.2
Aerosil^®^ 200	9.1	9.1	9.1	9.1	9.1	13.0	9.1	8.7	8.7
Methocel™ E5	9.1	9.1	9.1	9.1	9.1	8.7	9.1	8.7	8.7
Lactochem^®^ Crystals	-	-	-	24.2	-	-	-	-	-
Mannogem^®^ Powder	-	12.1	-	-	24.2	23.2	-	-	-
Mannogem^®^ XL	-	-	16.1	-	-	-	24.2	-	-
Kollidon^®^ CL	-	-	-	-	-	-	-	4.3	-
Explotab^®^ CLV	-	-	-	-	-	-	-	-	4.3

**Table 2:** Printing settings used for prototype cores.

Printhead	Printhead type	DD-135H
	Nozzle diameter (mm)	0.5
	Lift nozzle (mm)	0.5
	Feed rate (mm/s)	2
	Feed pressure (MPa)	0.100–0.500
Layer	Strand width (mm)	0.5
	Layer height (mm)	0.5
	Outer perimeter loops (n)	2
	Top/Bottom layers (n)	0
Infill	Infill density (%)	45
	Infill pattern type	Rectilinear
	Fill angle (°)	45
	Overlapping perimeter (%)	5

**Table 3:** Printing settings used for core-in-shell systems.

Printhead type	HPD-100 (Core)	DD-135H (Inner Shell)	DD-135H (Outer Shell)
Nozzle diameter (mm)	0.5	0.5	0.5
Lift nozzle (mm)	0.5	0.5	0.5
Feed rate (mm/s)	2	2	2
Feed pressure (MPa)	-- *	0.300–0.400	0.300–0.400
Strand width (mm)	0.5	0.5	0.5
Layer height (mm)	0.5	0.5	0.5
Outer perimeter loops (n)	0	2	2
Top/Bottom layers (n)	0	0	0
Infill density (%)	65	100	100
Infill pattern type	rectilinear	rectilinear	rectilinear
Fill angle (°)	45	45	45
Overlapping perimeter (%)	5	5	5

* Set as piston velocity.

## Data Availability

Dataset available on request from the authors.
